# Intersection of Sphingolipid and Sterol Metabolism at the Level of Orm Proteins in Yeast

**DOI:** 10.3390/cells15090814

**Published:** 2026-04-30

**Authors:** Francesca Barone, Stéphanie Cottier, Jiri Stribny, Michele Visentin, Roger Schneiter, Museer A. Lone

**Affiliations:** 1 Department of Clinical Pharmacology and Toxicology, University Hospital Zurich, University of Zurich, 8952 Zurich, Switzerland; francesca.barone@unical.it (F.B.); michele.visentin@usz.ch (M.V.); 2 Department DiBEST (Biologia, Ecologia, Scienze della Terra) Unit of Biochemistry and Molecular Biotechnology, University of Calabria, 87036 Arcavacata di Rende, Italy; 3 Department of Biology, University of Fribourg, Chemin du Musée 10, 1700 Fribourg, Switzerland; stephanie.cottier@unifr.ch (S.C.); jiri.stribny@unifr.ch (J.S.); roger.schneiter@unifr.ch (R.S.); 4 Institute of Clinical Chemistry, University Hospital Zurich, University of Zurich, 8952 Zurich, Switzerland

**Keywords:** sterols, sphingolipids, serine-palmitoyltransferase (SPT), steryl esters, neutral lipid mobilization, membrane microdomains, terbinafine, inositol auxotrophy

## Abstract

Sterols and sphingolipids assemble into specialized membrane microdomains that are essential for membrane function, protein sorting, and signal transduction. Although coordinated regulation between sterol and sphingolipid metabolic pathways has long been recognized, the molecular mechanisms mediating this cross-talk remain incompletely defined. Here, we uncover an unanticipated role for the conserved yeast Orm proteins in controlling sterol and neutral lipid homeostasis. Deletion of *ORM1* and *ORM2* causes hypersensitivity to sterol biosynthesis inhibitors, accumulation of steryl esters, and an increase in lipid droplet number. Consistent with mutants lacking core neutral lipid hydrolases, *orm1Δ orm2Δ* cells display a marked defect in neutral lipid mobilization. These phenotypes depend on sphingolipid pathway perturbation but cannot be attributed to sphingolipid accumulation alone. Together, these findings position the Orm proteins as regulatory nodes linking sterol metabolism, lipid droplet dynamics, and sphingolipid biosynthesis.

## 1. Introduction

Serine-palmitoyltransferase (SPT) catalyzes the condensation of serine and palmitoyl-CoA to form long-chain bases (LCBs), the amino-alcohol backbones that define all sphingolipids. As the first and rate-limiting enzyme in sphingolipid biosynthesis, SPT serves as a key regulatory hub whose activity is modulated by several interacting partners. In *Saccharomyces cerevisiae*, the SPT complex comprises the catalytic subunits Lcb1 and Lcb2. The conserved ORMDL protein family (Orm1/2 in yeast; ORMDL1/2/3 in mammals) negatively regulates SPT activity [1,2,3]. Additional components, such as Tsc3 and the phosphatidylinositol-4-phosphate (PI4P) phosphatase Sac1, physically associate with the super-complex [1,4,5]. However, SPT-Orm-Tsc3 complexes with or without Sac1 (SPOT and SPOTS complexes) are likely to coexist in vivo, representing alternate assemblies that modulate SPT activity and thereby sphingolipid homeostasis. Interestingly, yeast Orm1/2 possess N-terminal phosphorylation sites that are absent in the mammalian ORMDLs [1,6]. These N-terminal regions are dynamically phosphorylated by the AGC kinase Ypk1, linking Orm function to sphingolipid homeostasis [6].

Like sphingolipids, sterol metabolism is also tightly regulated in yeast and mammals to maintain membrane homeostasis and support essential processes including signaling, endocytosis, and transport of solutes [7,8,9]. Transcriptional control of sterol biosynthetic genes is mediated by the transcription factors Upc2 and Ecm22 in yeast and by SREBP2 in mammals. Sterols are esterified by acyl-CoA:cholesterol acyltransferases (Are1/2 in yeast; ACAT1/2 in mammals) and stored in lipid droplets (LDs) as steryl esters (STE) [10]. Hydrolysis of STE liberates free sterols to meet acute cellular demands [11], such as during rapid cell proliferation [10]. Thus, sterol biosynthesis, uptake, storage, and mobilization operate in a dynamic equilibrium. Despite parallels in their regulatory logic, the mechanistic links between sphingolipid and sterol metabolism remain poorly defined.

Genetic and biochemical evidence supports a coordinated regulation of sphingolipid and sterol metabolism [7]. For example, perturbation of yeast sterol biosynthetic enzymes induces marked remodeling of sphingolipid species [12,13]. In mammalian cells, elevated cholesterol levels trigger autophagy-mediated degradation of ORMDLs, leading to enhanced SPT activity [14]. Notably, impairing sphingolipid biosynthesis does not reciprocally alter the abundance or composition of yeast sterols [13]. Nonetheless, structural analyses of the yeast SPT complex have revealed direct binding of ergosterol to Orm and Lcb1 subunits, strongly suggesting that this constitutes a molecular interface between sterol and sphingolipid metabolism [4].

Here, we show that yeast *ORM* deletion mutants exhibit defects in sterol homeostasis. Importantly, although these alterations are influenced by sphingolipid pathway perturbation, our data indicate that they are not simply a consequence of elevated LCB/sphingolipid levels.

## 2. Materials and Methods

### 2.1. Strains, Media and Growth Conditions

Strains used in this study are listed in Table 1. Strains were grown in YPD rich medium [1% Bacto yeast extract, 2% bacto peptone (US biological, Swampscott, MA, USA), 2% glucose (Reactolab, Servion, Switzerland)] or synthetic complete media. Triple- and sextuple-lipase mutant strains were generated by sequential gene disruption using PCR-based deletion cassettes, with selectable markers recycled via a Cre/loxP recombination system, as described previously [15].

### 2.2. Sterol and Neutral Lipid Analysis by Thin Layer Chromatography (TLC)

For quantification of steady-state lipids, cell equivalent to 20 OD_600_ grown in synthetic complete medium were harvested. Lipids were then extracted with methanol:methyl-tert-butyl-ether:dichloromethane (4:3:3, *v*/*v*/*v*; MMD) containing cholesterol butyrate as an internal standard, for 1 h at 37 °C. Single-phase lipid extracts were obtained by centrifugation at 16,000× *g* for 5 min at room temperature. The supernatant was dried under N_2_ stream and reconstituted in chloroform. Samples were loaded on a HPTLC Silica gel 60 plate with a concentrating zone (Merck KGaA, Darmstadt, Germany) using an automated Camag ATS4 TLC sampler (Muttenz, Switzerland). Lipids were separated by one-dimensional thin layer chromatography (TLC) in n-hexane:n-heptane:diethyl ether:acetic acid (62.4:18.3:18.3:1, *v*/*v*/*v*/*v*) solvent. Staining was performed in 9.6% orthophosphoric acid (*v*/*v*) containing 3% copper acetate (*w*/*v*), and then the lipids were charred at 130 °C for 30 min. Lipid bands were visualized and quantified by fluorescence scanning at 366 nm in a Camag TLC Scanner 3 (Muttenz, Switzerland). Quantification was performed relative to the cholesterol butyrate internal standard and normalized to OD_600_ units.

### 2.3. BODIPY Staining of LD

LD visualization with BODIPY staining was performed as described previously [16]. Cells were incubated with BODIPY 493/503 (Invitrogen, Carlsbad, CA, USA) at a final concentration of 1 µg/mL for 30 min in the dark. Cells were washed once with 50 µM BSA (fatty acid free) in PBS and once with PBS only and re-suspended in residual PBS. Fluorescence microscopy was performed using a Carl Zeiss Axioplan 2 microscope (Carl Zeiss, Oberkochen, Germany) fitted with an AxioCam CCD camera and AxioVision software, version 3.1.

### 2.4. Neutral Lipid Mobilization

For neutral lipid mobilization experiments, strains were grown in YPD medium at 24 °C. An amount of 10 OD_600_ units of cells were pelleted down, resuspended in 1 mL of YPD and labeled with 5 µCi of [^3^H]-palmitic acid for 4 h; then, 9 mL of YPD was added and labeling was continued overnight. The following morning, cultures were centrifuged, and cells were re-suspended in 30 mL of YPD medium. To induce neutral lipid mobilization, cerulenin and terbinafine were used to final concentrations of 15 µg/mL and 30 µg/mL, respectively. An amount of 6 mL of culture was withdrawn at each time point, centrifuged at 4000 rpm for 5 min. Cell pellets were washed once with 1 mL of sterile water and frozen after adding 600 µL of chloroform/methanol (1:1, *v*/*v*) and equal volume of acid washed glass beads. Lipid extraction and analysis were performed as described previously [11]. Radioactivity in lipid extracts was quantified by scintillation counting. Equivalent counts were dried down under N_2_. Lipids were then resolved by TLC on silica gel 60 plates (Merck, Darmstadt, Germany) using petroleum ether/diethyl ether/acetic acid (70:30:2, *v*/*v*/*v*) as the mobile phase. Labeled lipids were quantified by linear scanning with a Berthold Tracemaster 40 automatic TLC analyzer. For mobilization experiments with long-chain bases, PHS (5 µM) was added to overnight-labeled cells 2 h prior to induction of neutral lipid mobilization (i.e., before addition of cerulenin and terbinafine) and was maintained throughout the assay following dilution.

### 2.5. Drug Sensitivity Test

For testing the sensitivity of different strains to sterol biosynthesis inhibitory drugs, cells were grown in YPD medium. Cultures were normalized to OD_600_ = 1, serially diluted tenfold and stamped on YPD plates containing different drugs: fenpropimorph (1.25 µg/mL) and terbinafine (30 µg/mL). Plates were incubated at 30 °C for 4 days.

### 2.6. Plasmid Generation

*ORM1* and *ORM2* expression constructs were generated by homologous recombination in yeast using the pRS426 (2µ, URA3) multicopy vector. For *ORM1*, the coding sequence together with 500 bp upstream (promoter region) and 404 bp downstream sequences was amplified using primers ORM1 pRS Clon_F (5′-GCGTAATACGACTCACTATAGGGCGAATTGGGTACCGGGCCGTAGGGCCGCCAGCGCCACCTGTC-3′) and ORM1 pRSClon_R (5′-TAGAACTAGTGGATCCCCCGGGCTGCAGGAATTCGATATCGCAATGTATACATTGGCAACTTGGC-3′).

For *ORM2*, the coding sequence together with 500 bp upstream and 429 bp downstream sequences was amplified using primers ORM2 Clon_F (5′-GCGTAATACGACTCACTATAGGGCGAATTGGGTACCGGGCGATTAAATTTAGGGTCCCCGGCATTG-3′) and ORM2 Clon_R (5′-TAGAACTAGTGGATCCCCCGGGCTGCAGGAATTCGATATCGCGTTTGCCATGATCTACCCTAGTG-3′).

PCR products were co-transformed with linearized pRS426 into yeast cells to enable in vivo recombination and plasmid assembly. Correct assembly was verified by colony PCR using vector specific primer pRS426_F (5′-ATGTGCTGCAAGGCGATTAAGTT-3′) and ORM1_Ctrl_R (5′-TAATGACCACATGAATAATCCAAGCGC-3′) for *ORM1* or ORM2_Ctrl_R (5′-CGAAGGTTTCCTGTTCCACATGTGA-3′) for *ORM2*. Plasmids were subsequently extracted from yeast and transformed into *E. coli* as described [17].

### 2.7. ESI-MS Analysis of Long-Chain Bases

Exponentially growing cells cultured at 24 °C in YPD medium were used for lipid extraction. Lipid extraction was carried out by a two-step lipid extraction protocol as described in [18] with some minor modifications. An amount of 20 OD_600_ units of cells were collected and washed once with sterile water. Cells were re-suspended in 1 mL of 150 mM ammonium bicarbonate (NH_4_HCO_3_) and 600 µL of glass beads added. Cell lysis was carried out using a Precellys 24 homogenizer (Bertin Technologies, Montigny-le-Bretonneux, France) at 5000 rpm, 3× 30 s on–30 s off. The cell lysate was diluted to 5 mL of 150 mM NH_4_HCO_3_.

LCBs were extracted with 10 mL of chloroform:methanol (17:1, *v*/*v*) containing C17-sphinganine (d17:0; Avanti Polar Lipids, Alabaster, AL, USA) as an internal standard for 2 h at 4 °C. After centrifugation at 700× *g* for 10 min, the lower organic phase was recovered and dried under N_2_ flow. Dried lipid extracts were dissolved in 100 µL of chloroform:methanol (1:2, *v*/*v*) containing 5 mM ammonium acetate. ESI-MS analysis was performed on a Bruker Esquire HCT ion trap mass spectrometer (Bruker, Billerica, MA, USA) by direct infusion (180 µL/h) in positive ion mode at 250 V capillary tension. Long-chain bases were identified based on their precursor *m*/*z* values and characteristic MS/MS fragmentation patterns following isolation of the precursor ions, including [M + H -H_2_O]^+^ and [M + H -2H_2_O]^+^ ions. Ion fragmentation was induced by argon (8 mbar). DHS (d18:0;2) was identified as the protonated ion [M + H]^+^ at *m*/*z* 302.2 with diagnostic fragments at *m*/*z* 284.2 and 266.2, whereas PHS (t18:0;3) was identified as the protonated ion [M + H]^+^ at *m*/*z* 318.3 with sequential dehydration fragments at *m*/*z* 300.3 and 282.3, consistent with commercially available standards (Avanti Polar Lipids, Alabaster, AL, USA). Identification of vinyl ether LCB [M + H]^+^ at *m*/*z* 344.3 was reported previously [19].

### 2.8. Molecular Docking Simulations

Molecular docking of ergosterol to SPOT-Orm complexes was performed using AutoDock Vina [20,21]. Cryo-EM-derived structures of the SPOT-Orm1 (PDB 8C81) [4] and SPOT-Orm2 (PDB 8QOG) [22] complexes were used as macromolecular targets. Ergosterol was obtained from PubChem (CID #444679) and converted to PDB format using PyMOL (version 2.5.4; Schrödinger, LLC, New York, NY, USA). Protein and ligand structures were prepared for docking using AutoDock Tools (version 1.5.7; The Scripps Research Institute, La Jolla, CA, USA), including assignment of polar hydrogens and conversion to PDBQT format [23]. Docking grids were defined to encompass the transmembrane region of the SPOT-Orm complexes. For control simulations, modified receptor structures were generated by either removing the N-terminal α1 and α2 helices of Lcb1 from the SPOT-Orm1 structure or modeling these helices into the SPOT-Orm2 structure based on the SPOT-Orm1 complex. Predicted docking poses were analyzed and visualized using UCSF ChimeraX (version 1.10.1; UCSF Resource for Biocomputing, Visualization, and Informatics, San Francisco, CA, USA) [24].

### 2.9. Statistical Analysis

Statistical analyses and graphs were generated with GraphPad Prism 10.0 (GraphPad Software, Inc., San Diego, CA, USA). *p*-values were calculated from one-way analysis of variance followed by Tukey’s post hoc test. Differences were considered statistically significant at *p* < 0.05.

## 3. Results

### 3.1. Yeast ORM Mutants Are Hypersensitive to Sterol Biosynthesis Inhibition

Yeast *ORM* mutants were previously shown to be sensitive to several chemical agents, including dithiothreitol (DTT) and tunicamycin [3]. Consistent with this, we previously showed that *ORM* mutants display an upregulated unfolded protein response (UPR) [2]. In addition, a high-throughput chemical–genetic screen, which profiled the fitness of yeast deletion mutants against over 400 small molecules and environmental stresses, identified hypersensitivity of *orm1Δ* and *orm2Δ* mutants to inhibitors of sterol biosynthesis, such as fenpropimorph and lovastatin [25]. Although azole antifungals (e.g., fluconazole, itraconazole) targeting the lanosterol 14α-demethylase Erg11 were included in the screen, no hypersensitivity was reported for *orm1Δ* or *orm2Δ* mutants to these compounds.

To independently validate this phenotype, we compared the growth of serially diluted wild-type (WT), single, and double *ORM* mutants on rich medium (YPD) containing sterol biosynthesis inhibitors. Terbinafine inhibits the fungal squalene epoxidase Erg1, blocking the conversion of squalene to lanosterol [26], whereas fenpropimorph is a potent inhibitor of the Δ^8^–Δ^7^ sterol isomerase Erg2 and the Δ^14^ sterol reductase Erg24 [27].

**Figure 1:**
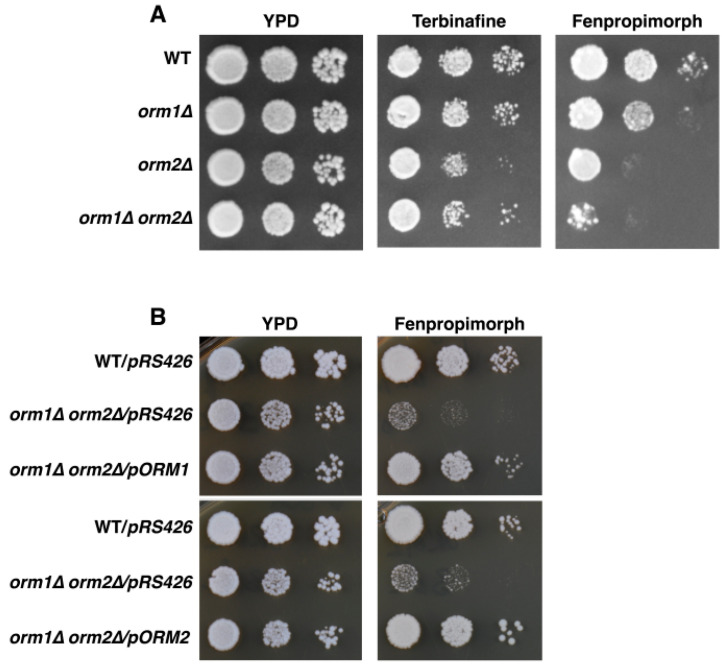
*ORM* mutants are hypersensitive to sterol biosynthesis inhibitors. (**A**) Growth defects in response to sterol biosynthesis inhibitors. Ten-fold serial dilutions of WT, *orm1Δ*, *orm2Δ*, and *orm1Δ orm2Δ* cells were spotted on YPD plates supplemented with terbinafine (30 µg/mL) or fenpropimorph (1.25 µg/mL) and incubated at 30 °C to assess drug sensitivity. (**B**) Plasmid-borne expression of *ORM1* or *ORM2* was tested for complementation of the fenpropimorph-sensitive phenotype. Growth of *orm1Δ orm2Δ* cells transformed with p*ORM1* or p*ORM2* was compared with the cell carrying the empty vector control (pRS426) under the same conditions.

On control plates, *ORM* mutants grew comparably to WT cells (Figure 1A). Terbinafine caused relatively mild growth inhibition of *orm2Δ* and *orm1Δ orm2Δ* double mutant cells, whereas fenpropimorph more strongly inhibited the growth of these cells (Figure 1A). On both drugs, *orm1Δ* cells exhibited a less pronounced phenotype than *orm2Δ* mutants (Figure 1A).

To confirm that the observed sensitivity is specific to *ORM* deficiency, we tested whether ectopic expression of *ORM* genes could rescue the drug sensitivity of *orm1Δ orm2Δ* cells. To this end, *ORM1* and *ORM2* were cloned into a multicopy pRS426 vector (2µ origin, URA3) together with their endogenous promoter and terminator sequences. Expression of either *ORM1* or *ORM2* restored growth of *orm1Δ orm2Δ* cells on fenpropimorph (Figure 1B). Notably, *ORM2* expression conferred a stronger rescue than *ORM1*.

### 3.2. ORM Mutants Accumulate Steryl Esters

**Figure 2:**
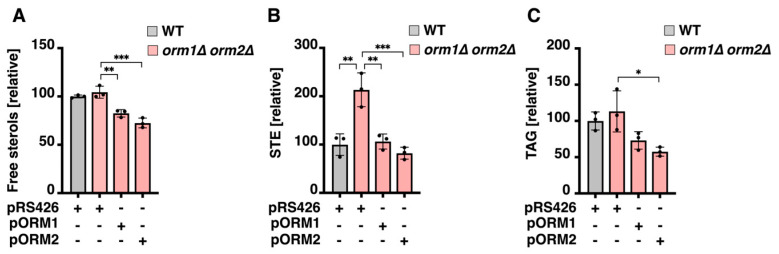
Neutral lipid accumulation in *ORM* mutants. (**A**–**C**) Quantification of free sterols (**A**), steryl esters (STE; **B**), and triacylglycerols (TAG; **C**) in WT and *orm1Δ orm2Δ* cells bearing an empty vector (pRS426) or complemented with p*ORM1* or p*ORM2*. Data represent mean ± SD (*n* = 3); * *p* < 0.05, ** *p* < 0.01, *** *p* < 0.001.

Alterations in sterol/ergosterol metabolism may underlie the growth inhibition observed on fenpropimorph-containing plates. To assess this possibility, steady-state levels of free sterols and STE were quantified in ORM-deficient yeast cells. Total free sterol content was comparable between WT and *orm1Δ orm2Δ* cells (Figure 2A). In contrast, *orm1Δ orm2Δ* cells exhibited a significant accumulation of STE (Figure 2B). STE, together with triacylglycerols (TAG), constitute the major neutral lipids stored in yeast LD. TAG levels, quantified from the same TLC analysis, were similar in WT and *orm1Δ orm2Δ* cells (Figure 2C). Complementation with either Orm1 or Orm2 restored STE levels to those of WT cells (Figure 2B). Interestingly, overexpression of Orm1 or Orm2 in the double mutant background further decreased both free sterol and TAG levels (Figure 2A,C), with the effect more pronounced upon Orm2 overexpression.

### 3.3. ORM Mutants Show Defective Neutral Lipid Mobilization

**Figure 3:**
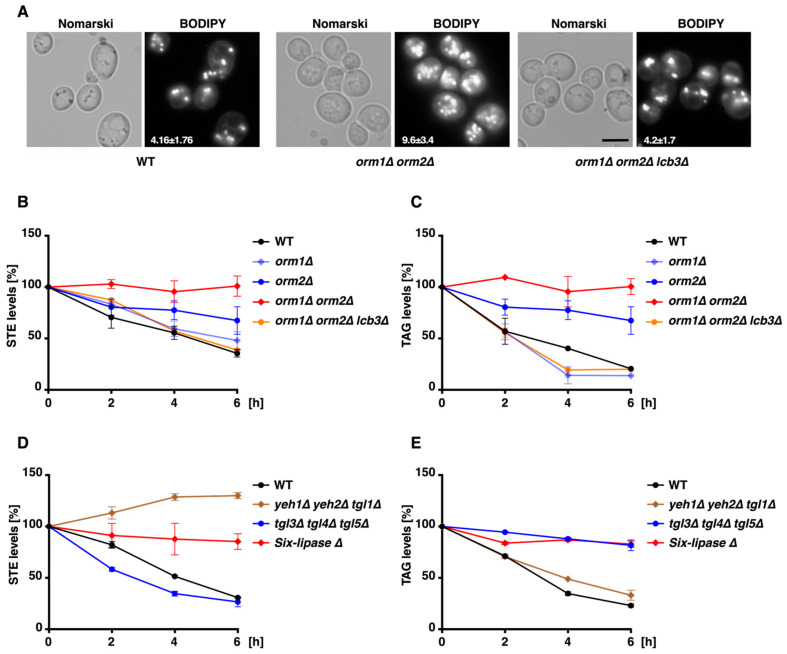
*ORM* mutants exhibit lipid droplet accumulation and defective mobilization of STE and TAG. (**A**) Neutral lipid staining and lipid droplet quantification. WT, *orm1Δ orm2Δ*, and *orm1Δ orm2Δ lcb3Δ* cells were stained with the neutral lipid dye BODIPY 493/503, and lipid droplet numbers per cell were quantified from fluorescent microscopy images. Scale Bar: 5 µm. (**B**,**C**) STE and TAG mobilization in *ORM* mutants. Mobilization of steryl esters (STE; **B**) and triacylglycerols (TAG; **C**) in WT, *orm1Δ*, *orm2Δ*, *orm1Δ orm2Δ*, and *orm1Δ orm2Δ lcb3Δ* cells following pulse labeling with [^3^H]-palmitic acid and chase in glucose-rich medium containing cerulenin and terbinafine. At the indicated time points, samples were collected, and lipids were extracted, separated by TLC, and quantified by radio scanning. Mobilization profiles of STE (**D**) and TAG (**E**) in hydrolase-deficient strains: *yeh1Δ yeh2Δ tgl1Δ* (STE hydrolase-deficient), *tgl3Δ tgl4Δ tgl5Δ* (TAG lipase-deficient), and the sextuple lipase mutant analyzed by [^3^H]-palmitic acid pulse-chase and TLC. The sextuple mutant phenocopied *orm1Δ orm2Δ*, failing to mobilize both neutral lipid classes. Data represent mean ± SD (*n* = 2).

To visualize STE related changes, WT and *orm1Δ orm2Δ* cells were stained with the neutral lipid-specific dye BODIPY 493/503. Consistent with the biochemical data, quantification of the fluorescence microscopy images showed a marked increase in the average number of LD per cell in the *orm1Δ orm2Δ* mutant (9.6 ± 3.4) compared with WT cells (4.1 ± 1.76) (Figure 3A).

Yeast *LCB3* encodes the sphingosine-1-phosphate phosphatase that is required for incorporation of exogenous LCB into sphingolipids [28,29,30]. LCB3 deletion alleviates tunicamycin sensitivity and restores protein export defects or UPR levels associated with *orm1Δ orm2Δ* cells [2]. Interestingly, *LCB3* deletion reduces PHS accumulation in *orm1Δ orm2Δ* cells [2]. Consistent with these observations, BODIPY staining in *orm1Δ orm2Δ lcb3Δ* cells also showed reduced average LD numbers (4.2 ± 1.7) (Figure 3A).

Given the specific increase in STE levels in *orm1Δ orm2Δ* cells and their heightened sensitivity to sterol synthesis inhibitors, we hypothesized that the mutants are defective in STE hydrolysis. To test this hypothesis, we examined the ability of *ORM* mutant cells to mobilize neutral lipids. Stationary-phase cultures of WT and *ORM* mutant cells were pulsed with [^3^H]-palmitic acid and subsequently diluted into YPD medium containing 2% glucose, terbinafine, and the fatty acid synthesis inhibitor cerulenin [31]. Although terbinafine mildly inhibits growth of *ORM* mutants (Figure 1A), the concentrations used here, combined with cerulenin, inhibit de novo sterol and fatty acid synthesis without completely halting growth, thereby forcing cells to mobilize STE and TAG stores to supply sterols and fatty acids for membrane expansion during the assay period.

In these experiments, *orm1Δ* cells mobilized STE similarly to WT cells, while *orm2Δ* cells showed reduced mobilization and the *orm1Δ orm2Δ* double mutant failed to mobilize STE (Figure 3B). Surprisingly, TAG mobilization was also impaired in both *orm2Δ* and *orm1Δ orm2Δ* cells (Figure 3C). Additionally, *LCB3* deletion restored both STE and TAG mobilization patterns in the *orm1Δ orm2Δ* cells (Figure 3B,C).

In yeast, Yeh1, Yeh2, and Tgl1 function as the major STE hydrolases, while Tgl3, Tgl4, and Tgl5 serve as the principal TAG lipases [11,31,32]. Consistent with this, under our conditions, the *yeh1Δ yeh2Δ tgl1Δ* triple mutant failed to mobilize STE (Figure 3D), whereas *tgl3Δ tgl4Δ tgl5Δ* cells were blocked in TAG mobilization (Figure 3E). A sextuple mutant lacking all six lipases failed to mobilize both STE and TAG, similar to what was observed in the *orm1Δ orm2Δ* double mutant (Figure 3D,E).

### 3.4. Long-Chain Base Accumulation Alone Does Not Account for Defective Neutral Lipid Mobilization in orm1Δ orm2Δ Cells

**Figure 4:**
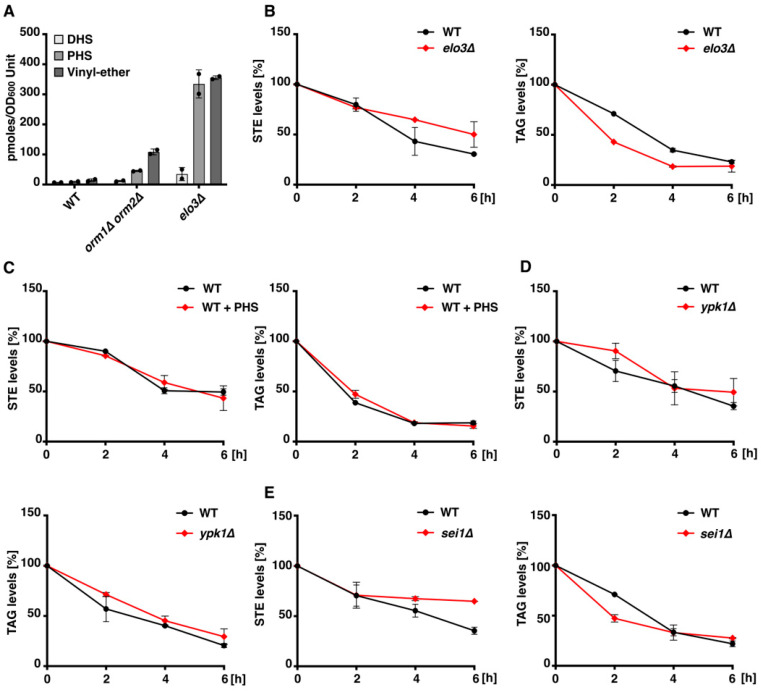
Elevated long-chain bases and altered lipid droplet morphology do not impair neutral lipid mobilization. (**A**) Quantification of long-chain bases. Levels of dihydrosphingosine (DHS), phytosphingosine (PHS), and PHS vinyl ether quantified by ESI-MS in WT, *orm1Δ orm2Δ*, and *elo3Δ* cells. (**B**) Neutral lipid mobilization in *elo3∆* cells. Mobilization of STE (**left**) and TAG (**right**) in WT and *elo3Δ* cells following [^3^H]-palmitic acid pulse-chase. (**C**) Effect of exogenous PHS on neutral lipid mobilization in WT cells. STE and TAG mobilization was unaffected in WT cells supplemented with 5 µM PHS. (**D**) Neutral lipid mobilization in *ypk1Δ* cells. STE and TAG mobilization in *ypk1∆* cells compared with WT under pulse-chase conditions. (**E**) Neutral lipid mobilization in *sei1Δ* (seipin-deficient) cells. STE and TAG mobilization in *sei1∆* cells, which exhibit aberrant LD morphology, shows no impairment relative to WT. Data represent mean ± SD (*n* = 2).

We asked whether PHS/sphingolipid accumulation acts as a general modulator of neutral lipid mobilization. In budding yeast, the most abundant ceramide and complex sphingolipid species contain phytosphingosine (PHS; t18:0) conjugated to hydroxylated and non-hydroxylated C26 very long-chain fatty acids (VLCFAs) [18]. Elo3, an endoplasmic reticulum (ER)-localized 3-ketoacyl-CoA synthase, generates C26-VLCFA [33]. *ELO3* deletion reduces levels of VLCFA-containing sphingolipids while increasing those with shorter fatty acids [18,33]. Moreover, accumulated PHS in *orm1Δ orm2Δ* and *elo3Δ* cells is converted to a PHS vinyl ether [20]. In any case, levels of dihydrosphingosine (DHS), PHS, and the PHS vinyl ether are higher in the *elo3Δ* cells than in the *orm1Δ orm2Δ* cells (Figure 4A). Despite this, neutral lipid mobilization was comparable between WT and *elo3Δ* cells under our conditions (Figure 4B).

Yeast cells efficiently import and assimilate exogenous LCBs supplied in the growth medium [34]. We therefore assessed neutral lipid mobilization in WT cells in the presence of PHS. Supplementation with PHS (5 µM), however, did not affect TAG or STE mobilization in WT cells (Figure 4C).

We next tested whether regulators upstream of the ORMs influence this pathway. Ypk1, which inhibits Orm proteins to activate SPT and the ceramide synthase subunit Lac1, functions as a key pacesetter of sphingolipid synthesis in yeast [6,35]. However, *ypk1Δ* mutant cells showed normal neutral lipid mobilization activity (Figure 4D). Additionally, we investigated whether the aberrant LD morphology in *orm1Δ orm2Δ* cells contributes to defective neutral lipid mobilization. Although deletion of the Berardinelli-Seip congenital lipodystrophy type 2 homolog (Sei1 in yeast; seipin in mammals) alters LD morphology and promotes sphingolipid accumulation in yeast and mammalian cells [36,37,38], this phenotype differs from that in *orm1Δ orm2Δ* cells. Nevertheless, neutral lipid mobilization in WT and *sei1Δ* cells was comparable (Figure 4E), suggesting that LD morphology changes alone are insufficient to impair mobilization.

### 3.5. Defective Neutral Lipid Mobilization in Sac1 Deletion Mutants

**Figure 5:**
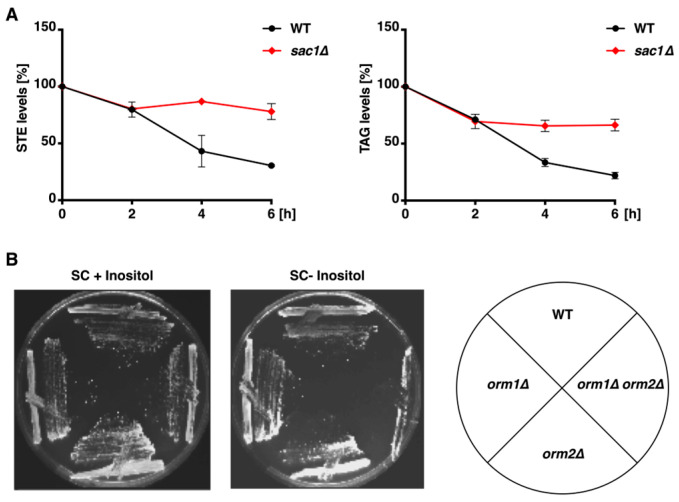
Shared defects in neutral lipid mobilization and inositol auxotrophy in *sac1∆* and *ORM* mutants. (**A**) Impaired neutral lipid mobilization in *sac1∆* cells. Mobilization of TAG (**left**) and STE (**right**) in WT and *sac1Δ* cells following pulse-chase labeling with [^3^H]-palmitic acid. *sac1Δ* mutants show impaired mobilization of both lipid classes. Data represent mean ± SD (*n* = 2). (**B**) Inositol auxotrophy of *ORM* mutants. Growth of WT, *orm1Δ*, *orm2Δ*, and *orm1Δ orm2Δ* strains on synthetic complete (SC) medium with (+) or without (−) inositol showing that *orm1Δ orm2Δ* cells exhibit inositol auxotrophy.

LD accumulation has previously been reported in temperature-sensitive *sac1^ts^* [39] and s*ac1Δ* cells [38]. Although Sac1 and Orm proteins do not directly interact within the SPT complex [1,4], concomitant deletion of *SAC1* and *ORM1/2* results in synthetic lethality [1]. We therefore assessed neutral lipid mobilization in *sac1Δ* cells. Under our conditions, both TAG and STE mobilization were compromised in *sac1Δ* cells (Figure 5A). The *sac1Δ* mutant cells exhibit inositol auxotrophy [39,40]. Consistent with the previous reports [2], *orm1Δ orm2Δ* cells also displayed inositol auxotrophy (Figure 5B). These data support the notion that yeast Orm1/Orm2 and Sac1 function in parallel converging pathways.

### 3.6. In Silico Sterol Binding Analysis to SPT Complexes

**Figure 6:**
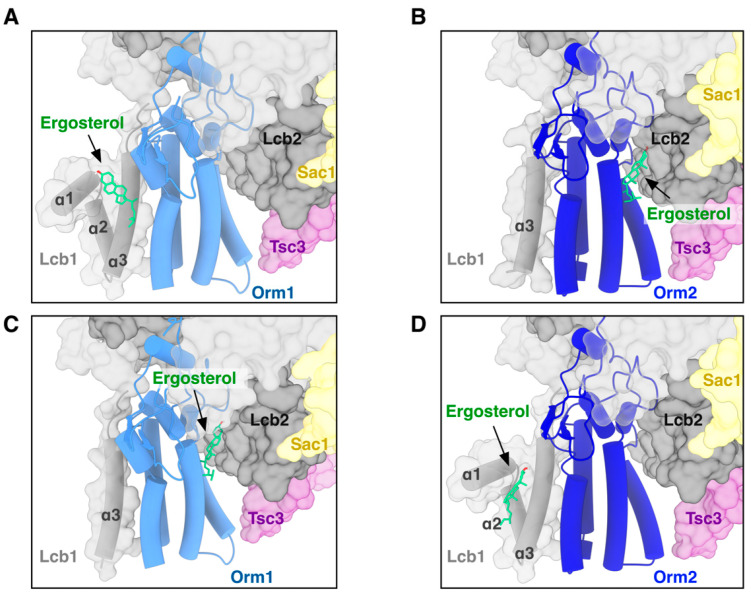
Simulations of ergosterol accommodation in SPT-Orm1/2 complexes. (**A**) Molecular docking simulation of ergosterol (green) into the SPOT-Orm1 complex positions the sterol within a pocket formed by the N-terminal α1–α3 helices of Lcb1 and transmembrane helices of Orm1, consistent with previously reported structural observations (PDB 8C81, ref. [4]). (**B**) Docking of ergosterol into the cryo-EM structure of the SPOT-Orm2 complex (PDB 8QOG, ref. [22]), which lacks resolved N-terminal α1 and α2 helices of Lcb1, results in sterol positioning at an alternative interface between Orm2 and Lcb2. (**C**) Control docking simulation in which the N-terminal α1 and α2 helices of Lcb1 were removed from the SPOT-Orm1 structure, resulting in relocation of ergosterol to a site similar to that observed in panel (**B**). (**D**) Control docking simulation of the SPOT-Orm2 complex in which the N-terminal α1 and α2 helices of Lcb1 were modeled based on the SPOT-Orm1 structure, shifting ergosterol docking toward the interface involving the Lcb1 N-terminal helices and Orm2.

Orm2 deletion cells exhibited stronger growth inhibition in response to sterol biosynthesis inhibitors and more pronounced defects in STE mobilization than Orm1 mutants. In contrast, direct ergosterol binding has been resolved only for the Orm1-containing SPOTS complex [4]. The available cryo-EM structure of the Orm2-containing SPT complex lacks the resolved N-terminal helices of Lcb1 that form the sterol-binding interface in the Orm1 complex [22], precluding direct assessment of sterol engagement in this assembly. To assess whether this apparent difference reflects a genuine inability of Orm2-containing complexes to bind sterols or instead results from incomplete structural information, we employed molecular docking as a hypothesis-generating approach. Docking simulations recapitulated the previously described ergosterol-binding pocket in the SPOTS-Orm1 complex (Figure 6A). When ergosterol was docked into the cryo-EM structure of the SPOT-Orm2 complex, the sterol adopted alternative positions at interfaces involving Orm2 and Lcb2 (Figure 6B). To directly test whether the presence or absence of the Lcb1 N-terminal helices dictates sterol positioning, we performed control docking simulations in which these helices were either removed from the SPOT-Orm1 structure or modeled into the SPOT-Orm2 structure. Removal of the Lcb1 N-terminal helices from the Orm1 complex redirected ergosterol away from the canonical binding pocket to sites similar to those observed in the Orm2 structure (Figure 6C). Conversely, modeling the Lcb1 N-terminal helices into the Orm2 complex shifted ergosterol docking toward the canonical interface observed in the Orm1-containing complex (Figure 6D). These in silico analyses support the notion that Orm2-containing complexes are structurally capable of accommodating ergosterol.

## 4. Discussion

In this study, *ORM* deletion mutants exhibit defects in sterol/STE metabolism, including STE accumulation, increased LD numbers, and impaired neutral lipid mobilization, phenocopying strains lacking core LD hydrolases.

Previous work has shown that perturbations in LD biogenesis or breakdown can modulate complex sphingolipid levels, particularly in strains lacking TAG lipases (*tgl3Δ tgl4Δ tgl5Δ*) [41,42]. We observed that *orm1Δ orm2Δ* cells display pronounced alterations in sterol and neutral lipid metabolism (Figure 1 and Figure 2A–C). *LCB3* deficiency, which restores PHS levels in the *orm1Δ orm2Δ* mutant cells [2], also rescued LD number and neutral lipid mobilization (Figure 3A–C). These data suggest that sphingolipid accumulation may contribute to the observed neutral lipid phenotypes. However, other observations argue against sphingolipid accumulation being the sole driver of mobilization defects observed in the *orm1Δ orm2Δ* cells: (i) the *elo3Δ* mutant accumulates higher levels of LCBs, yet mobilizes neutral lipids normally (Figure 4A,B); (ii) supplementation with exogenous PHS does not impair mobilization (Figure 4C); and (iii) *ypk1Δ* and *sei1Δ* mutants, despite sphingolipid metabolism or LD morphology defects, mobilize TAG and STE normally (Figure 4D,E). Together, these observations may indicate that ORMs influence sterol and neutral lipid metabolism through a specific sphingolipid-dependent metabolic or signaling axis. This is conceptually consistent with recent findings in mammalian systems. In human and murine endothelial cells, pharmacological or genetic ablation of S1P synthesis increases SPT activity via an S1P receptor-ORMDL feedback axis [43]. However, further studies are required to determine how modulation of sphingolipid metabolism through ORMs, as opposed to mechanistically distinct perturbations such as Elo3 deletion, is coupled to or uncoupled from neutral lipid mobilization.

However, a selective increase in steady-state levels of STE, without a corresponding increase in TAG, suggests that *ORM* deletion may differentially impact the biosynthetic pathways or regulatory feedback mechanisms for sterols versus TAG. For instance, potential reductions in phospholipid synthesis reported in *orm2Δ* mutants [44] could limit fatty acid availability for TAG production, preventing its accumulation despite impaired mobilization.

The hypersensitivity of *orm2Δ* and particularly *orm1Δ orm2Δ* mutants to terbinafine and fenpropimorph further supports the presence of a sterol-handling defect. The observed growth inhibition could result from several non-mutually exclusive factors: (i) an insufficient free sterol pool due to impaired STE hydrolysis for rapid membrane expansion, (ii) impaired trafficking of sterols from the ER, (iii) defective transport/activation of enzymes, such as LD-associated lipases, and, lastly, (iv) disrupted interactions between Orm proteins and components of the sterol biosynthetic machinery.

Sterols and sphingolipids are highly enriched at the plasma membrane, where they form ordered lipid raft domains. Although their biosynthesis is coordinated, crosstalk appears asymmetric: sterol defects remodel sphingolipid profiles, whereas sphingolipid mutants generally spare sterol composition [13]. In this context, the neutral lipid mobilization defects observed in *ORM* mutants suggest that Orm proteins integrate sterol metabolism with SPT regulation, consistent with direct binding of ergosterol by SPT [4]. Orm proteins may therefore function as sterol-responsive regulators that couple local sterol availability to ER-LD communication.

Yeast Orm2 has a more pronounced effect on sphingolipid accumulation than Orm1 [2]. Additionally, the differential sensitivities of *orm1Δ* and *orm2Δ* mutants to sterol inhibition underscore functional divergence between the paralogs. However, the rescue observed upon plasmid-based expression for *ORM1* may additionally reflect differences in gene dosage associated with the multicopy nature of the vector.

Our in silico docking models (Figure 6) are consistent with the ergosterol binding capacities of Orm-containing SPT complexes. Absence of experimentally resolved sterol density in the Orm2 structure likely reflects structural heterogeneity or incomplete resolution rather than an intrinsic inability to engage sterols. Consequently, the stronger phenotypic impact of Orm2 may reflect differences in regulatory capacity, such as expression dynamics, phosphorylation status, or interactions within ER membrane domains. Independent large-scale datasets identify both genetic and physical interactions between Orm2 and enzymes of the ergosterol biosynthesis pathway. Orm2 exhibits negative genetic interactions with Erg2, Erg3, and Erg24, and shows physical proximity to sterol demethylation enzymes, including the C14 demethylase Erg11 and the C4 demethylation components Erg25 and Erg26 [45,46,47]. Taken together, these findings support a model in which Orm2 functions as a sterol-responsive regulatory node at the ER membrane, coupling sphingolipid and sterol homeostasis in a manner not fully shared by Orm1.

Consistent with prior reports of LD accumulation in *sac1Δ* cells [25,26], we observed pronounced defects in TAG and STE mobilization in these mutants (Figure 6). The synthetic lethality between *sac1Δ* and *orm1Δ orm2Δ* points [1] to a critical functional interplay between Sac1 and Orm proteins. This interaction likely reflects their roles in partially redundant or parallel pathways that converge to maintain lipid homeostasis. Given that Sac1 also regulates SPT activity through PI4P turnover [1,5], it is conceivable that altered PI4P signaling provides a mechanistic bridge linking sterol metabolism, LD function, and SPT regulation. Elucidating this connection could reveal how spatial lipid signaling coordinates membrane organization and storage lipid utilization within the ER network.

**Figure 7:**
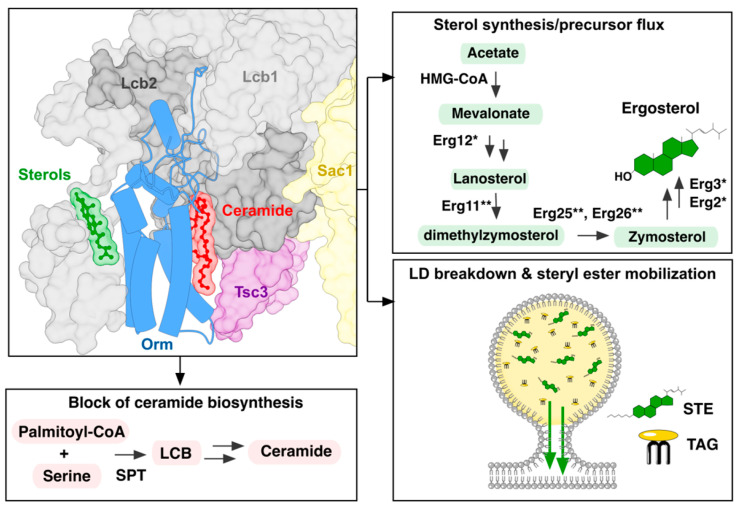
Model for the integration of sterol and neutral lipid metabolism through SPOT-Orm activity. Working model summarizing the role of Orm-containing complex at the intersection of long-chain base (LCB)/sphingolipid biosynthesis by the serine-palmitoyl transferase (SPT), sterol homeostasis, and lipid droplet (LD) metabolism. The SPOT-Orm complex is adapted from PDB 8C81 (ref. [4]). * and ** depict enzymes in sterol biosynthesis pathway that show genetic or physical interaction with yeast Orm proteins.

These findings reveal an unexpected role for Orm proteins beyond their established function as negative regulators of SPT, positioning ORMs at a regulatory intersection between sphingolipid biosynthesis, sterol homeostasis, and LD function (Figure 7). Together, our data reveal an unappreciated role for Orm proteins in integrating sterol availability, LD mobilization, and sphingolipid biosynthesis into a coordinated regulatory network. While the capacity of Orm proteins to bind sterols directly is unlikely to be influenced by a specific genetic background, we cannot exclude that the magnitude of the sterol and lipid droplet phenotypes may vary depending on genetic context. Future studies extending these findings to additional strain backgrounds and mammalian ORMDL proteins will be important to further establish the generality of this regulatory axis. Nevertheless, this functional interplay underscores the broader significance of Orm-mediated lipid control for maintaining cellular lipid balance. Given the evolutionary conservation of ORMDL proteins and their emerging roles in human metabolic and neurological disorders, dissecting these mechanisms in yeast offers a valuable framework for understanding how perturbations in lipid flux contribute to pathology.

## Figures and Tables

**Table 1:** Yeast strains used in this study.

Strain	Genotype	Source
BY4742	*MAT*α; *his3∆1*, *leu2∆0*, *lys2∆0*, *ura3∆0*	Euroscarf
RSY5189	*[BY4742] pRS426*	This study
HXX1-7D	*[BY4742] orm2::Kan^r^*, *orm1::clonNAT^r^*	Han et al., 2010 [2]
ACX144-1B	*[BY4742] orm2::Kan^r^*, *orm1::clonNAT^r^*, *lcb3::Kan^r^*	Han et al., 2010 [2]
RSY5191	*[HXX1-7D] pRS426*	This Study
RSY5389	*[HXX1-7D] pRS426-* *ORM1^prom^-ORM1*	This study
RSY5390	*[HXX1-7D] pRS426-* *ORM2^prom^-ORM2*	This study
RSY4206	*[BY4742]* yeh1*::LoxP*, yeh2*::LoxP*, tg11*::LoxP*	This study
RSY4205	*[BY4742] tgl3::LoxP*, *tgl4::LoxP*, *tgl5::LoxP*, *yeh1::LoxP*, *yeh2::LoxP*, *tgl1::LoxP*	This study
RSY4389	*MATA; his3∆1*, *leu2∆0*, *met15∆0*, *ura3∆0* *tgl3::LoxP*, *tgl4::LoxP*, *tgl5::LoxP*	This study
*elo3∆*	*[BY4742] elo3::KanMX*	Euroscarf
*ypk1∆*	*[BY4742] ypk1::KanMX*	Euroscarf
*sei1∆*	*[BY4742] sei1::KanMX*	Euroscarf
*sac1∆*	*[BY4742] sac1::KanMX*	Euroscarf

## Data Availability

The original contributions presented in this study are included in the article. Further inquiries can be directed to the corresponding author.
